# Acute Hepatocellular Drug Induced Liver Injury Probably by Alfuzosin

**DOI:** 10.1155/2015/101062

**Published:** 2015-02-22

**Authors:** Tufan Cicek, Huseyin Savas Gokturk, Gulhan Kanat Unler

**Affiliations:** ^1^Department of Urology, Baskent University, Hocacihan Mahallesi Saray Caddesi No. 1, Selcuklu, 42080 Konya, Turkey; ^2^Department of Gastroenterology, Baskent University Faculty of Medicine, Konya, Turkey

## Abstract

Alpha blockers are the drugs that exert their effects by binding to alpha receptors and relaxing smooth muscles and are currently used for treatment of benign prostate hyperplasia (BPH). These drugs are often tolerated well by the patients. However, they also possess some common side effects. Hepatotoxicity, on the other hand, is quite rare. We report herein a case with the rare complication of acute hepatocellular drug induced liver injury (DILI) by administration of Alfuzosin.

## 1. Introduction

Alpha blockers are currently used for treatment of benign prostate hyperplasia (BPH). These drugs exert their effects by binding to alpha receptors and relaxing smooth muscles. They are used for alleviating symptoms of urinary system disorders [1]. There are 5 types of alpha blockers that are in general use. These are Tamsulosin, Doxazosin, Silodosin, Terazosin, and Alfuzosin. These drugs are often tolerated well by the patients. However, they also possess some common side effects. These include asthenia, vertigo, impotence, and postural hypotension [1]. Hepatotoxicity, on the other hand, is quite rare. It has been reported only in a few patients after Terazosin and Alfuzosin use in the literature [2–5].

We report herein a case with the rare complication of acute hepatocellular drug induced liver injury (DILI) by administration of Alfuzosin.

## 2. Case Report

A 65-year-old male patient presented to the urology department of our hospital with the complaints of prostatism in January 17, 2014. His prostate-specific antigen level was 0.796 ng/ml. Uroflowmetry was performed, which revealed a reduced urine flow rate, a prolonged micturition time, and obstructive findings. The patient was begun on Alfuzosin prolonged-release tablets at a dose of 10 mg given perorally as a single dose. The patient presented to the urology department at the 9th day of therapy with the loss of appetite and malaise initially, followed by itching on whole body, darkening of urine, and yellowish discoloration of eyes. He gave no history of aspirin, paracetamol, or herbal medicine use or a history of hepatic disease. The patient's liver tests were normal before the treatment, and Alfuzosin was discontinued. On physical examination there were no stigmata of liver disease. Laboratory test results were as follows: leukocyte count: 5.8 K/*μ*L (4.5–11 K/*μ*L), aspartate aminotransferase (AST): 175 U/L (0–40 U/L), alanine aminotransferase (ALT): 402 U/L (0–55 U/L), alkaline phosphatase (ALP): 235 U/L (15–250 U/L), gamma glutamyl transferase (*γ*-GT): 327 U/L (8–61 U/L), total bilirubin: 7.6 mg/dL (0.2–1.2 mg/dL), and direct bilirubin, 5.8 mg/dL (0–0.25 mg/dL). Ultrasonography showed normal liver, gall bladder, common bile duct, and intrahepatic bile ducts. Serological examinations ruled out acute viral hepatitis with negative levels of HBsAg, anti-HBc IgM, anti-HCV, anti-HAV Ig M, anti-HEV, HEV-RNA, and HCV RNA. The patient was also examined in terms of autoimmune hepatitis (ANA, AMA, ASMA, and LKM-1) and metabolic liver diseases (transferrin saturation, ferritin, alpha-1 antitrypsin, ceruloplasmin, 24-hour urinary Cu^2+^, and Kayser-Fleischer ring in eye examination) but all tests were normal. EMA was negative and serum IgA was normal. In order to evaluate intrahepatic bile ducts the patient was examined with magnetic resonance cholangiopancreatography (MRCP) that revealed a normal result. His RUCAM (Roussel UCLAF causality assessment method) score was 7 (probable adverse reaction to drug). The medical therapy was stopped and the patient was monitored. Intermittent laboratory tests demonstrated gradual normalization of transaminases and bilirubin levels (Table 1).

## 3. Discussion

We aimed to report a patient who had no risk factors for or signs of liver disease and was taking no drugs or substances apart from Alfuzosin, known or suspected to cause acute hepatocellular DILI. Alfuzosin is a quinazoline derivative used in treatment of BPH. It is a selective *α*_1_ blocker. It is metabolized in liver and the inactive metabolites are excreted in feces. The use of the drug has been linked to dizziness, respiratory infections, headache, and fatigue [1]. However, Alfuzosin has a very favorable safety profile. It has been approved by FDA for use in symptomatic BPH. It may also cause GI complaints, albeit to a lesser degree. However, Alfuzosin-induced hepatotoxicity is substantially rare [3–5].

DILI is one of the most common causes of liver injury, which can develop following the use of a variety of drugs through a number of mechanisms. This is because liver is the organ where metabolism of many drugs and chemicals takes place. Thus, almost every medical therapy has a hepatotoxicity potential [4]. The clinical picture of DILI can be highly dependent on the culprit chemical and varies from asymptomatic elevation of liver enzymes to fulminant hepatic failure. The clinical picture of toxic hepatitis may mimic any acute or chronic liver disease and the most common symptoms are jaundice, fatigue, loss of appetite, nausea, and vomiting. Our patient also presented with similar complaints. The injury may have a course characterized by a hepatocellular injury, with elevation of aminotransferase levels or by a cholestatic injury, with elevated alkaline phosphatase levels (with or without hyperbilirubinemia) as the main finding. Based on the biochemical abnormalities, our case had a hepatocellular type of liver injury [6]. Increased transaminases and bilirubin levels together with symptoms beginning following the onset of therapy made us consider acute DILI. Other etiologies were excluded with imaging modalities and laboratory tests. Our diagnosis was confirmed by normalization of laboratory tests as well as a marked improvement in the clinical picture following discontinuation of the culprit drug.

The most important factor in diagnosis of DILI is a high index of suspicion. It is essential to take a detailed history and perform a thorough physical examination. The past history should include questioning about NSAID, acetaminophen, and long-term antibiotic use. The differential diagnosis should include alcoholic cirrhosis, blood transfusion, and all forms of liver diseases. Imaging modalities such as ultrasonography or MRI should be used both for diagnosis and differential diagnosis [7].

The etiology of Alfuzosin-induced hepatotoxicity is unknown. It is considered to be idiosyncratic. DILI may become manifest as acute, chronic, or fulminant hepatitis. All etiological agents with a hepatotoxicity potential should be excluded by a gastroenterologist using both clinical and laboratory data. The diagnosis of DILI requires a time window of 1 week to 3 months following the use of the drug, normalization of liver function tests after discontinuation of the responsible drug, and recurrent liver damage following reinstitution of the drug. However, literature data suggest that the duration of drug use has varied between 3 and 36 weeks in previous reports [3, 5]. The corresponding duration was 9 days in our patient. Two previous publications reported a mixed cholestatic and hepatocellular injury in one patient and an acute hepatocellular injury in the other [3, 5]. An acute hepatocellular injury developed following drug use in our patient who had no history of liver disease. Compared to previous reports, our patient experienced the shortest time period between the onset of drug therapy and symptom onset and also between the discontinuation of the drug and laboratory and symptomatic improvement [3, 5]. Liver biopsy can be used to confirm the diagnosis and exclude other differential diagnoses. However, a liver biopsy is usually not required for diagnosis of most cases of DILI. We did not perform a liver biopsy. As far as we know, there are only three other reports of severe hepatotoxicity related to Alfuzosin use. Our case is the fourth case of DILI by Alfuzosin. Urologists should definitely question their patients in terms of previous liver disease before putting patients on Alfuzosin therapy. Patients developing liver test abnormalities should definitely be managed and followed by a gastroenterologist.

## Figures and Tables

**Table 1:** Laboratory parameters.

Laboratory parameters	Pre-Alfuzosin	February 17 (Alfuzosin)	February 25	January 12
ALT (0–55 U/L)	10	402	60	41
AST (0–40 U/L)	23	175	132	32
*γ*-GT (8–61 U/L)	9	327	36	53
ALP (15–250 U/L)	15	235	198	133
T. bilirubin (0.2–1.2 mg/dL)	0	7.6	8.9	7.9
D. bilirubin (0–0.25 mg/dL)	0	5.8	6.7	5.6
